# Respiratory Syncytial Virus's Non-structural Proteins: Masters of Interference

**DOI:** 10.3389/fcimb.2020.00225

**Published:** 2020-05-19

**Authors:** Elena Margaret Thornhill, David Verhoeven

**Affiliations:** Veterinary Microbiology and Preventive Medicine, Iowa State University, Ames, IA, United States

**Keywords:** nonstructural (NS) proteins, respiratory syncitial virus, antiviral pathway, innate immunity, mitochondria

## Abstract

Respiratory Syncytial Virus (RSV) is a highly prevalent virus that affects the majority of the population. The virus can cause severe disease in vulnerable populations leading to high hospitalization rates from bronchiolitis or secondary bacterial infections leading to pneumonia. Two early and non-structural proteins (Ns1 and Ns2), strongly over-ride the antiviral innate system but also diminish the adaptive response as well. This review will cover interactions of Ns1 and Ns2 with the host antiviral response with a focus on alterations to signaling pathways, cytokine gene expression, and effects of the Ns proteins on mitochondria.

## Introduction

Respiratory Syncytial Virus (RSV) is a highly prevalent and infectious virus. Moreover, most individuals have contracted the virus by the age of one, and half of all children over the age of two have contracted RSV at least twice (Glezen et al., 1986). RSV causes almost 60,000 hospitalizations of children under 5 years of age in the U.S. alone and is responsible for 10,000 geriatric U.S. deaths each year (Falsey and Walsh, 2005; Rose et al., 2018). In adults, RSV infection often mimics that of the common cold, with symptoms occasionally reaching the severity of flu, but is often more severe in infants and the elderly (RSV Symptoms Care, 2018). Severe RSV infection can result in bronchiolitis, which is inflammation of the bronchioles in the lung and is the main reason children need hospitalization. RSV infection also results in a high number of secondary bacterial infections (Heikkinen et al., 1999; Weinberger et al., 2015). Moreover, bacterial pneumonia is attributable to RSV in 20.3% of children aged 1 year or younger and in 10.1% of children aged 1 to 2 years old. In contrast, bacterial pneumonia attributable to influenza infection occurs in only 3.2% of children aged 1 to 2 years (Weinberger et al., 2015). RSV is the primary catalyst for secondary ear infections, with RSV, detected in the middle-ear fluid of 48 of the 65 children (74%) with acute otitis media (Heikkinen et al., 1999). RSV is a fascinating virus impacting large portions of the population and yet knowledge about the viral life cycle and regulation is limited. Importantly, there is still no licensed vaccine for RSV despite the decades of attempts highlighting a need for better understanding the viral pathogenesis and mechanisms fostering immunity.

## Viral Biology and Cell Antiviral Pathway Activation

**Figure 1:**
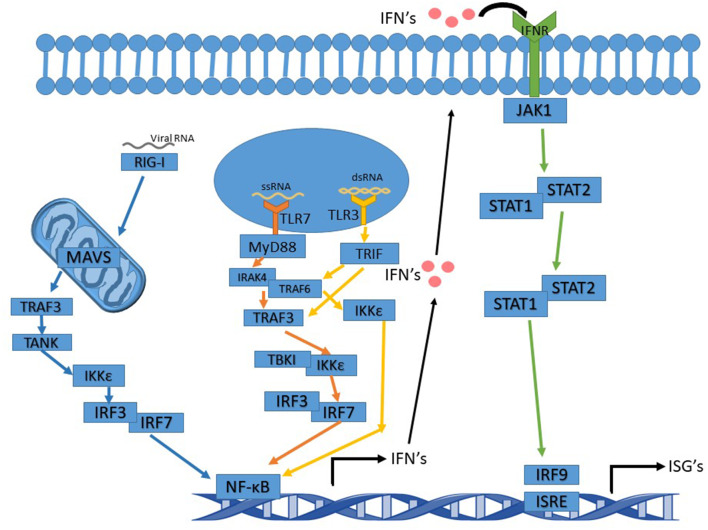
Interferon signaling pathway altered by Ns. TLR7 pathway's arrows are in orange and activation leads to activation of NF-κB and IFN induction. TLR3 pathway's arrows are in yellow. Activation of TRIF by TLR3 can either activate TRAF3 or TRAF6. Whichever one is activated leads to slight variations in the cascade. The IFNR pathway's arrows are in green and the RIG-I pathway is in blue.

RSV is an enveloped negative-sense single-stranded RNA virus of the family Pneumoviridae and the order Mononegavirales. RSV, and like other RNA viruses, triggers the toll-like receptor (TLR) and the RIG-I (Retinoic acid-inducible gene I) pathways upon infection to initiate a signal cascade that leads to the production of type 1 interferons (IFN). This activation pathway is summarized in Figure 1. While RSV proteins or RNAs interact with multiple TLR's including TLR2 and TLR4, the TLR's that are involved in eliciting the interferon response, like many other RNA viruses, are TLR3 and TLR7.

TLR3, upon binding to dsRNA (double-stranded RNA), becomes activated and recruits TRIF (TIR-domain-containing adapter-inducing interferon-β), which proceeds to initiate a signal cascade that activates TRAF3 and TRAF6 (TNF receptor-associated factors). In turn, this activates IKK (IκB kinase). After activation of IKK by TRAF3, IRF3, and IRF7 (Interferon Regulatory Factors) are the next molecules to be activated in the pathway. This leads to the production of NF-κB (nuclear factor kappa-light-chain-enhancer of activated B cells) and the induction of type 1 interferon production. IKK activation by TRAF6 does not activate IRF3 or IRF7 but still induces a NF- κB response and leads to the production of type 1 interferons (Ning et al., 2011; Shin and Harris, 2011; Kawasaki and Kawai, 2014).

TLR7 binds to and is activated by ssRNA (single-stranded RNA), subsequently acting via recruiting MyD88 and signaling the cell through its pathway. MyD88 activates IRAK4 (Interleukin-1 receptor-associated kinase), which then activates IRAK1 activating TRAF6. TRAF6 activation in the MyD88 pathway leads to activation of IRF7, which then activates NF-κB and the induction of type 1 interferon production (Ning et al., 2011; Kawasaki and Kawai, 2014; Suthers and Sarantopoulos, 2017).

Type 1 interferons function as antiviral defense mechanisms and as a way of alerting other cells in the vicinity of an invader. Type I IFNs consist of seven classes, including IFNα and IFNβ. IFNα and IFNβ are produced by multiple cell types, though dendritic cells are the primary producer of IFNα. Type 1 interferons are produced first by the TLR or RIG-I pathways, and then fed into the JAK-STAT pathway when they are excreted from the producing cell and bound to an IFN receptor on the surface of the same or other cells (Pestka et al., 2004; Hervas-Stubbs et al., 2011; Ivashkiv and Donlin, 2014). In addition to type 1 IFN's, there are also type 2 IFN's known as IFNγ. IFNγ is crucial to RSV clearance by the immune system (Gonzalez et al., 2012). The RIG-I pathway feeds into the TLR pathways to activate type 1 interferon production. RIG-I activates MAVS (mitochondrial antiviral-signaling protein) which is located in/on the mitochondria. MAV activates TRAF3, which in turn activates TANK, which then activates IKKε leading to IRF3 activation. IRF3 then acts as it does in the TLR pathway (2014; Reikine et al., 2014; Zevini et al., 2017).

## Viral Replication and NS Protein Production

RSV has ten genes coding for 11 proteins, and in order, these genes are Ns1, Ns2, N, P, M, SH, G, F, M2 (−1,−2), and L. In order to undergo successful transcription, RSV requires its M2-1 protein, a transcription elongation factor, in addition to the N, P, and L proteins. Other members of the Mononegavirales order, such as its closest relatives metapneumovirus and parainfluenza, only require the N, P and L proteins for this process. During RSV transcription, the polymerase proceeds along with the transcript from the first start site, right before the Ns1 gene, in a sequential, termination-re-initiation (stop-start) mechanism (Kuo et al., 1997). RSV genes are under one transcriptional promotor (Collins and Wertz, 1983) and can initiate transcription at either a +1 or a +3 initiation site before the NS1 start site. The RdRp (RNA dependent RNA polymerase) can initiate transcription independently at both positions. Initiation at either the +1 or the +3 site determines whether transcription or replication will occur. Initiation at the +3 site yields a short leader transcript (~25nt) and then reinitiates at the gene start site (gs) immediately preceding Ns1 to produce mRNA's. Initiation at the +1 site leads to full-length transcripts that are readily encapsidated by N (Tremaglio et al., 2013; Noton et al., 2019).

Since the viral genes must be transcribed in order, the order of genes in the genome is vital in the timing of infection. Ns1 and Ns2 (non-structural proteins) are the first genes to be transcribed. These proteins, which are essential for permissive *in vivo* infection, function to inhibit the type 1 interferon (IFN) response, other elements of the immune system, and may contribute to the high incidence of secondary bacterial infections in RSV infections due to its interference with the immune system (Teng and Collins, 1999; Jin et al., 2000; Bossert and Conzelmann, 2002; Lo et al., 2005; Elliott et al., 2007; Ling et al., 2009; Verhoeven et al., 2014; Sun and Lopez, 2017). Thus, it is imperative that Ns1/Ns2 are made early during infection (Jin et al., 2000; Collins et al., 2013). The non-structural genes of RSV are unique to the virus, as they have low, if any homology, with genes of other viruses in the family serving a similar function (Swedan et al., 2009; Wu et al., 2012). This review will further focus on the non-structural genes of RSV, the interactions of their proteins, and their importance in the viral life cycle of RSV.

## Ns1 vs. Ns2

Of RSV's non-structural genes, Ns1 has been more studied more in-depth, and thus more of its role in infection is known than that of Ns2. In terms of immune suppression, Ns1 is the dominant player, and Ns2 may be a minor one, though important still for enhancing and supporting RSV pathogenesis. RSV's Ns1 is 139 amino acids long and Ns2 is slightly smaller at 124 amino acids (Atreya et al., 1998) (GenBank: AFM95347.1, AFM95348.1). Ns1 is the first gene in the genome and follows a 44-nt leader region thus making it the most abundant transcripts produced. NS1 is followed directly by Ns2 with a small intergenic region of around 19nts spacing between the two open reading frames (Atreya et al., 1998).

While the Ns1 protein is fairly stable after translation and can be detected in pulse chase experiments for hours, the Ns2 protein disappears rapidly in the early infection stage and is thought to have an intracellular half-life of 1hr. Both Ns proteins have been observed in multimeric forms which are thought to be linked by disulfide bridges, though the predominant form of Ns1 is thought to be its monomeric form (Collins et al., 1996; Evans et al., 1996; Chatterjee et al., 2017). Ns1 and Ns2 can form homo- and heteromers. Ns1 traffics to the nucleus, while Ns2 as well as a Ns1-Ns2 complex traffic to the mitochondria. Most of the Ns1 and Ns2 in an infected cell resides in the mitochondria as a heteromer (Swedan et al., 2011). Furter study is needed to explain why the majority of the Ns proteins reside here, though it may be a way to sequester them as a means of regulation; as Ns1 inhibits viral replication including in minigenome systems (Atreya et al., 1998) and mitochondrial metabolic byproducts and proteins play critical roles in the antiviral response (Koshiba, 2013; Jin et al., 2017; Mohanty et al., 2019). While being sequestered in the mitochondria may appear counterintuitive, the Ns1 protein is subject to post-translational modification, which may indicate an additional regulatory mechanism (Atreya et al., 1998). This modification occurs by proteolytic cleavage, producing a 12.5K protein that is thought to be produced shortly after the production of the normal-sized Ns1. It is unknown what function the modification has and may be a byproduct of Ns1. As the intensity of the Ns1 band in pulse-chase experiments decreased the 12.5 K protein appeared. While Ns1 is thought to be relatively stable, the discrepancies in different papers may indicate that only certain forms of the protein are stable (Huang et al., 1985; Collins et al., 1996). The Ns genes inhibit the type 1 interferon response as well, so this may allow for a pause in viral replication until IFN production is inhibited and RSV can replicate without being suppressed by the innate antiviral response. STAT1 and IFNAR1 knockout mice have increased RSV titer/load compared to wildtype mice through all recorded time points (Hashimoto et al., 2005; Goritzka et al., 2014). RSV has an unusual replication growth curve, with slower and less productive replication and gene expression compared to other viruses of the same genome type (Atreya et al., 1998). The inhibition of replication by Ns1 may help to explain this phenomenon, especially since it is the earliest and most abundant viral protein.

RSV has considerable variation in its genome between strains, and the amount of variation varies by the gene. Between the RSVA and B strains, the G gene has the most sequence variation. Ns1 and Ns2 have 22% nucleic acid differences between theses strains, with 13 and 8%, respectively differences in amino acid sequence. For comparison, the N gene which is thought to be very conserved differs by 14 and 4% for nucleotide and amino acid sequence similarity, respectively (Sullender, 2000). Comparing within the A strains from 2006 to 2019 against the prototypical A2 strain, the NS1 gene is fairly well-conserved on the amino acid level with about 97 or 100% identical amino acids to each other (using protein alignments). These amino acid changes, when they occur, do appear to occur within predicted MHCI binding peptides. Comparing NS2 strains between 2006 to 2019, the amino acid sequence is also very conserved with 98–100% conservation. Of interest, comparing mid/late 2,000 strains to earlier strains from the 1980s and some 1990 strains shows a substitution of R to K at the 38th amino acid. Scanning this protein with MHCI epitope binding prediction software (IEBD), this site falls within a conserved amino acid sequence that may bind to all the major MHCI within different ethnicities (i.e., HLA-A-2 and HLA-A-68). Some 1990s strains also have another change in another predicted MHCI epitope with a change from R to D at the 8th amino acid position. Going back to the sequence differences between A and B strains, most of the amino acid changes fall within predicted MHCI binding epitopes suggesting immune pressure between the A and B strains but not as much within each strain inside the same decade of circulation (i.e., 1–3 epitope changes between 2016 A and B strains in three predicted epitopes). These suggest a general lack of immune pressure on this protein as usually only one predicted CD8 T cell epitope changes but not necessary from 1 year to the following year. Thus, the amount of sequence conservation across strains may have relevance for possible drug therapies or vaccine targets, but may also have relevance for host range determination. The Ns genes may determine host range, as human RSV is capable of infecting bovine cells and vice versa though they are not capable of replicating in cows. When a virus containing the Ns genes of a non-native host (i.e., transfer of BRSV ns genes into HRSV) and infects non-native host cells, it replicates but is attenuated compared to the wild type (Bossert and Conzelmann, 2002).

The Ns proteins have multiple recorded interactions with cellular and other viral proteins. Ns1 has more recorded interactions and an enormous scope of impact when compared to Ns2. Ns1 is able to interact with Ns2, the RSV M protein, and is capable of inhibiting multiple parts of the immune system (Evans et al., 1996). Ns2, however, seems to be more limited in its inhibitory capacity as most data on its interaction record it only inhibiting the interferon response in infected cells. Together the Ns genes are capable of interfering with both the adaptive and innate antiviral immune response pathways (Atreya et al., 1998).

## Manipulation of Innate Antiviral Defense by NS Proteins

The Ns proteins are known to bind to and inhibit various molecules in the type 1 interferon response signal cascade of either the RIG-I or TLR pathways. Ns1 and Ns2 form degradosomes that home to the mitochondria, and are capable of degrading multiple proteins in the type 1 interferon pathway (Elliott et al., 2007; Swedan et al., 2009; Goswami et al., 2013). Ns1 and Ns2 have a curious relationship with the mitochondria as optimal degradosome function appears in modified mitochondria that are shorter and more motile. The modified mitochondria possess a greater surface area and thus greater access to MAVS (Goswami et al., 2013).

The Ns degradosome is a heterogeneous large protein complex 300–750 kD in size. The degradosome targets TRAF's (TNF receptor-associated factors), TBK1 (TANK Binding Kinase 1), RIG-I, IRF3/7, and STAT2. Through direct interaction with RIG-1, Ns2 suppresses RLR (RIG-I-like Receptor) signaling as well as prevents RIG-I from interacting with MAVS to prevent downstream signaling. While the exact composition of the degradosome is unknown, Ns1 acts as a functional E3 ligase and to facilitate proteasomal degradation of target proteins (Sun and Lopez, 2017). The helix α3 structure in the Ns1 protein, which is a highly conserved sequence across RSV strains, is thought to be of critical importance for the function of the protein. The helix α3 structure is important for the stability of the protein, IFN inhibition, as well as for the suppression of DC maturation (Chatterjee et al., 2017).

## RIG and TLR Interactions With NS Proteins

The RIG-I and TLR pathways both function to induce the production of interferons after contact with a PAMP (pathogen-associated molecular pattern), and feed into one another's pathways due to overlapping functions. An effect on one often, but not always, has an effect on the other. As stated previously, Ns1 is able to enter the mitochondria of cells. After entry, the protein can bind to and inhibits MAVS (Boyapalle et al., 2012) which serves as the intermediary protein downstream of RIG-I. The Ns genes also inhibit the proteins IRF-7, and IRF-3, which are involved in both the RIG-I and the TLR pathways (Lo et al., 2005; Elliott et al., 2007; Ling et al., 2009). RIG-I is essential for the IFN induction during RSV infection, similar to other RNA viruses such as influenza (Killip et al., 2015; Mäkelä et al., 2015; Wu et al., 2015). In contrast, Ns2 binds RIG-I directly and inhibits its functions (Lo et al., 2005; Elliott et al., 2007; Ling et al., 2009).

The type of cell that is infected by RSV seems to have an effect on which antiviral pathway is most helpful in limiting viral replication. The primary pathway used by dendritic cells to target and respond to an RSV infection is the TLR pathway, whereas lung epithelial cells use more of the RIG-I pathway as the primary form of response and clearance (Swedan et al., 2009). RSV infection is more prevalent in lung epithelial cells than in dendritic cells (Olszewska-Pazdrak et al., 1998; Zhang et al., 2002; Lukens et al., 2009; Rivera-Toledo and Gomez, 2012), and RSV's Ns proteins can target multiple stages of the TLR and RIG-I pathways. Ns1 can target TRAF3, and IKKε and leads to a reduction in the amounts of these proteins. TRAF3 intersects both the TLR and RIG-I pathways, and its inhibition allows RSV to evade the signaling caused by its production of viral RNA and its F protein (Swedan et al., 2009). Ns2 inhibits the activation of IRF3 early in infection as well as the activation of IFN via RIG-I. Ns2 interacts with RIG-I using the N-terminal 229aa of RIG-I, preventing RIG-I-MAVS interaction and preventing downstream activation of IRF3. Ns2 is able to both antagonize IFN induction and signaling, as well as inhibiting apoptosis, promoting continued viral replication (Bitko et al., 2007). Ns2 is able to bind to the N-terminal CARD (Caspase activation and recruitment domain) of RIG-I. This interaction inhibits RIG-I-MAVS interaction and prevents the downstream induction of the IFN gene in this pathway. Since RIG-I and IRF3, both of which are inhibited by Ns2, are important for the induction of type III interferons that this may be the mechanism by which RSV suppresses type III IFN's (Swedan et al., 2011; Barik, 2013). IFN-λ is a type III interferon which is associated with mucosal surfaces (Zanoni et al., 2017). In Ns deletion studies IFN-λ showed increased accumulation with the effect being most pronounced with deletion of both Ns1 and Ns2, though Ns1 deletion alone showed a statistically significant increase (Spann et al., 2004). As IFN-λ may prove to be the predominant IFN produced in RSV infection this is an exciting area of research worthy of more study (Okabayashi et al., 2011). According to Spann et al., deletion of Ns1 and Ns2 resulted in a 300-fold increase in the expression of IFN-α compared to mock-treated cells at 18hrs; the increase in expression decreased over time. Deletion of just Ns1 led to an 18-fold increase, with Ns2 deletion not showing a significant increase. Deletion of the Ns proteins also had an effect on IFN-β showing an increase of 40,000 fold for the double deletion at 18hrs (Spann et al., 2004). Ns2 is capable of weakly reducing the amount of TRAF3 while causing a slight increase in IKKε. Unlike Ns1, Ns2 is able to directly reduce STAT2 (Swedan et al., 2011; Goswami et al., 2013). The inhibition of STAT2 means that RSV has ways in which to inhibit not only the induction of the primary wave of type 1 interferon response but also the secondary (Lo et al., 2005; Elliott et al., 2007; Ling et al., 2009).

## STAT Pathways

The STAT pathway portion of the interferon system induces a way to respond to secreted interferons in an autocrine or paracrine fashion to induce a secondary wave of interferons. Not only is Ns1 able to inhibit the primary induction of IFN production, but it can degrade STAT2 through interactions with Ns2 and Elongin-Cullin E3 Ligase. It is also able to inhibit the secondary induction of IFN production via the IFN receptor signaling pathway (Lo et al., 2005; Elliott et al., 2007; Ling et al., 2009). To achieve this, Ns1 has multiple interactions involving the IFN receptor signaling pathway including but not limited to, upregulating SOCS1 and SOCS3 (suppressor of cytokine signaling), triggering STAT2 degradation, targeting the interferon-alpha receptor and inhibiting its response, and downregulating the JAK-STAT signaling pathway through STAT2 degradation (Xu et al., 2014; Zheng et al., 2015; Zhang et al., 2016). Multiple members of the paramyxovirus family, of which RSV used to be classified under until 2016, degrade STAT2 as a mechanism for inhibiting IFN1 production in infected cells. Mumps and Simian Virus 5 degrade STAT1, and human parainfluenza type 2 degrades STAT2. Both these mechanisms function to hamper IFN-α/β signal transduction (Elliott et al., 2007). Both Ns1 and Ns2 have a C-terminal tetrapeptide known as DLNP, which is involved in IFN suppressive functions like binding to MPA1B (microtubule-associated protein 1B) which is essential for the STAT2 reduction by Ns2 later on in infection (Swedan et al., 2011).

## Adaptive Immune System Manipulations

The Ns1 protein of RSV also plays a role in altering CD4+ (helper T cells) and CD8+ (cytotoxic T cell) cells. Ns1 suppresses the CD103+ CD8+ T cell (tissue-resident memory T cells) response, promote a Th2 (T helper cell) response, and suppress the Th17 response (Munir et al., 2011). Ns1 also increases the percentage of CD4+ T cells positive for IL-4 (interleukin) and enhances their proliferation by antagonizing the anti-proliferative effect of interferon type 1 (IFN-I). Munir et al. show that Ns1 depresses the concentration of IFNγ in DC-T cell co-cultures. The authors state that their data suggests that the Ns1 protein contributes to skewing of the Th1/Th2 balance toward Th2 during the priming of naive T cells or stimulation of memory T cells (Munir et al., 2011).

Deletion of the Ns1 protein results in an increase in dendritic cells (DC) maturation (Munir et al., 2008, 2011). While RSV induces the maturation of DC, these RSV-matured DC have an impaired ability to activate CD4+ T lymphocytes, possibly due to Ns1 and Ns2 mediated suppression of gene functions or mitochondrial mediators known for cell to cell signaling. Maturation can also be affected when Ns2 is also deleted, though Ns2 deletion alone does not yield a significant increase in DC maturation. Munir et al. measured DC maturation through cell surface expression and production of chemokines and cytokines. In the absence of RSV's Ns genes, the markers CD83 and CD38 were upregulated (Munir et al., 2008). It is thought that CD83 suppression by the Ns genes may explain the impaired activation of T-cells due to a reduction in costimulatory function and may lead to an impaired expansion of antigen-specific CD8+ cells. Suppression of CD38 reduces the survival of mature DC and results in a shift in T cell differentiation from favoring Th1 cells. The suppression of DC surface markers depends on the antagonism of the type 1 interferon response, as seen through the use of an IFNAR2-blocking monoclonal antibody (Munir et al., 2008). This antibody blocks the increase in surface markers and other effects of Ns gene deletion, and in the presence of the IFNAR2 blockade, there is also reduced RSV-induced maturation, indicating that IFN's can contribute to DC maturation despite the presence of the Ns genes (Munir et al., 2008).

## Ns Effect on Cell Cycle and Viral Replication

The Ns genes of RSV are crucial for the replication and proliferation of the virus in many ways. Prior studies suggest that the Ns genes are required for a permissive infection *in vivo* but are not required for a permissive infection *in vitro*, though these viruses grow more slowly (Teng and Collins, 1999; Jin et al., 2000; Bossert and Conzelmann, 2002). However, some researchers have found that while Ns1 deletion strongly attenuates the *in vivo* infection, the gene is not strictly required for permissive infection (Teng et al., 2000).

Additionally, Ns1 and Ns2 are capable of inhibiting premature apoptosis, which protects the virus's cellular resources allowing it to continue replicating. Several RSV proteins, including the Ns proteins, have been shown to block or delay apoptotic death of infected cells (Bitko et al., 2007). The mechanism behind this may be IFN independent as observed in A549 and Vero cell, perhaps through inducing the expression of anti-apoptotic factors like AKT (Barik, 2013; Sun and Lopez, 2017). Both Ns1 and Ns2 induce the production of anti-apoptotic molecules early in infection such as NF-κB, AKT, PDK, and GSK. Other viruses such as PIV5 (which is related to RSV) also use some of their proteins to inhibit apoptosis, indicating that this behavior may be typical in RNA viral IFN-antagonists (Barik, 2013). Ns1 is involved in modulating the cell cycle of the infected cell and is thought to be a key player during the G1-phase arrest. RSV infected cells, in general, seem to have a G0/G1-phase preference (Barik, 2013).

## Conclusion

**Figure 2:**
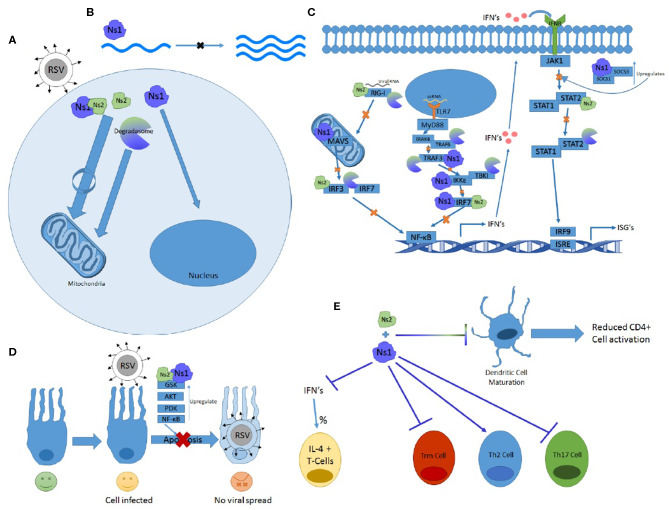
The many functions of Ns1 and Ns2. **(A)** Ns2 and Ns1-Ns2 complex home to the mitochondria where the majority of these proteins reside. The Ns formed degradosome also traffics to the mitochondria while Ns1 homes to the nucleus. **(B)** Ns1 inhibits viral replication. **(C)** Ns1, Ns2, and degradosome interactions with the RIG-I and TLR pathway showing which proteins and at what steps they inhibit IFN induction and signaling. **(D)** Ns1 and Ns2 upregulate anti-apoptotic proteins and prevent apoptosis. **(E)** Ns1 suppresses Trm and Th17 differentiation while promoting Th2 cell differentiation and the percentage of T cells expressing Il-4. Both Ns1 and Ns2 inhibit DC maturation.

The Ns genes serve an essential purpose in the lifecycle of RSV and have far-reaching implications for symptomology and pathology of the virus. They are capable of affecting almost every stage of the immune response and have multiple ways of effecting the same systems. From inhibiting both the primary and secondary waves of interferon production via TLR, RIG-I, and STAT pathways to altering immune cell maturation and differentiation, the Ns proteins prove crucial in blocking the immune response and protecting RSV from the antiviral response. Some of their features and functions outlined in this review are summarized in Figure 2.

Despite their crucial function of immune suppression, they are still considered to be unessential accessory proteins, which can be deleted *in vitro* with little effect of viral replication as a whole. *In-vivo* they may serve as possible vaccine deletion mutants or treatment targets as their deletion serves to attenuate the virus. They may also serve a purpose in suppressing anti-inflammatory treatments as Ns1 suppresses glucocorticoid receptor (GR) nuclear translocation and may block it completely (Xie et al., 2017). Ns1 interacts with IPO13 (importin) and may compete with GR for IPO13 binding, providing a mechanism of suppression or blockage. The lack of GR translocation may explain why RSV infection seems to lead to glucocorticoid insensitivity (Xie et al., 2017).

RSV infection in infants and the elderly are known to cause significant inflammation in the lungs, especially in the lower respiratory tract. Experiments in aged mice and cotton rats show that aged animals have a delayed viral clearance with reduced IFN-γ levels, as also observed in infants (Lee et al., 2005; Boukhvalova et al., 2007; Fulton and Varga, 2009; Verhoeven, 2019). IFN-γ is a significant factor in RSV clearance and is secreted by CD8+ and CD4+ T cells (Gonzalez et al., 2012). Moreover, Ns1 can alter CD8+ and CD4+ T cells and reduces the amount of IFN-γ produced by dendritic cells (Munir et al., 2011). Other sources of inflammation in RSV infection include the upregulation of TNF-α production through TLR activation and enhanced recruitment of neutrophils (Abu-Harb et al., 1999; McNamara et al., 2005; Rudd et al., 2005; Dou et al., 2013). While neutrophils are readily detected in infected airways CD8+ and CD4+ T cells needed to clear infection are often only detected at low levels (Rosenberg and Domachowske, 2012). Neutrophils recruitment by Il-8 is increased during RSV infection and can increase inflammation further damaging to the lung epithelia (Abu-Harb et al., 1999; McNamara et al., 2005). Neutrophils can be infected by RSV, and there is some evidence that RSV infection of neutrophils may inhibit their phagocytic ability (O'Donnell et al., 1998; Rohwedder et al., 1998; Torres et al., 2010; Halfhide et al., 2011; Verhoeven et al., 2014). It is unknown how this may be occurring, but it would not be surprising if the NS genes were involved in some way due to their ability to affect other immune cells.

The Ns proteins traffic to the mitochondria and are sequestered there but is unknown what they are doing while there. Perhaps they are further tweaking gene regulation. How and why the Ns genes specifically alter the expression and ratios of T helper and other immune cells also needs to be determined. While Ns1 inhibits replication in minigenome systems, it has not been shown to inhibit replication in other systems. Since Ns1 traffics to the nucleus, it may be inhibiting cellular transcription or replication there. The M and Ns1 proteins are closely associated, and M traffics to the nucleus where it inhibits cellar transcription, but these interactions in the nucleus need further study. More research needs to be done on the mechanisms of action of the Ns genes as their inhibitory effects on the immune system contribute to the severe symptomology associated with RSV infection. The timing of Ns function during in infection is not well-understood, and there is evidence that both genes may be modified and are inherently unstable with some reports showing loss of protein after 30 min (Huang et al., 1985; Collins et al., 1996; Evans et al., 1996). There is even some evidence that Ns2 maybe secreted which would allow for easier treatment targeting, or as an addition to a subunit vaccine strategy to limit overstimulation by the immune system (Collins et al., 1996).

Currently there are vaccine candidates under investigation that have Ns2 deletions, deletion of the Ns genes are attenuating with deletion on Ns1 having a greater effect than deletion of Ns2 and deletion of both is considered to be over attenuating. While promising both vaccine candidates combine other viral alterations with the Ns2 deletion indicating that Ns2 deletion alone may be under attenuating. A viable vaccine candidate for RSV that involves the deletion or modification of the Ns genes must be careful to strike the balance between over and under attenuation of the virus, which may be difficult given the lack of knowledge of the Ns genes (Jin et al., 2000; Bitko et al., 2007; Munir et al., 2008; Luongo et al., 2013; Karron et al., 2019). Through further study of the Ns genes and their activity in cells, we can better understand RSV and find novel therapies and vaccine strategies to better combat RSV in the future, especially given how common this virus is.

## Author Contributions

DV contributed to the writing and editing of the manuscript. ET wrote the manuscript.

## Conflict of Interest

The authors declare that the research was conducted in the absence of any commercial or financial relationships that could be construed as a potential conflict of interest.
